# Validity and reliability of the Chinese version of Threats of Artificial Intelligence Scale (TAI) in Chinese adults

**DOI:** 10.1186/s41155-023-00247-1

**Published:** 2023-02-21

**Authors:** Jie Cai, Zixuan Xu, Xiaoning Sun, Xiaojun Guo, Xurong Fu

**Affiliations:** 1 Department of Psychology, Zhejiang Sci-Tech University, Hangzhou, China; 2 Shanghai Children’s Medical Center, School of Medicine, Shanghai Jiao Tong University, Shanghai, China; 3 School of Education Science, Gannan Normal University, Ganzhou, China; 4 Institute of Mental Health, Nanjing Xiaozhuang University, Nanjing, China

**Keywords:** Threat perceptions, Artificial intelligence, Scale development, Validity, Chinese

## Abstract

With the outbreak of the COVID-19 pandemic, artificial intelligence (AI) has been widely used in fields such as medical treatment, while the threat of artificial intelligence has also received extensive attention. However, this topic has been only limitedly explored in China. To provide a measurement tool for AI threat research in China, this study aimed to examine the validity and reliability of the Threats of Artificial Intelligence Scale (TAI) in two Chinese samples of adults (*N1* = 654, *N2* = 1483). Results of exploratory factor analysis (EFA) and confirmatory factor analysis (CFA) suggested that the one-factor model of TAI as the best fitting model. Furthermore, the Chinese TAI was significantly related to Positive and Negative Affect Scale and Self-Rating Anxiety Scale, proving good criterion-related validity of the Chinese TAI. In sum, this study suggested the Chinese version of the TAI as a reliable and effective tool in assessing AI threat in the Chinese context. Limitations and future directions are discussed.

## Introduction

Artificial intelligence (AI) refers to a set of technologies that enable the systems to perceive, understand, react, and learn (Glikson & Woolley, 2020). It not only allows for automation, but also gives machines the ability to demonstrate mechanical, analytical, intuitive, and empathic intelligence (Huang & Rust, 2018). Previous studies showed that AI has brought changes to the society profoundly (Makridakis, 2017). For example, AI is now increasingly important in marketing due to increased computing power, availability of big data, and advances in machine learning algorithms and models (Huang & Rust, 2020). In addition, the researchers applied AI to the medical expert system and showed that the synarchy algorithm could be used to speed up the implementation, analysis, and evaluation of building models (Dadunashvili, 2018).

While advancing social changes, on the other hand, AI has not been fully utilized by the general public. One of the obstacles underlying such lack of general utilization may be the extent of user acceptance of AI (Bitkina et al., 2020), further attributable to potential AI-related threats perceived by the general population (Kieslich et al. 2021). For example, Liang and Lee (2017) found that a large number of Americans reported fear of autonomous robot and AI, which was different from the previously hypothesized human-technology relationship. Indeed, some researchers found evidence that threat perception played an important role in participants' technological aversion. That is, users who perceive AI as a high threat tend to have worse attitudes toward AI (Złotowski et al., 2017). More importantly, previous studies have shown that many people believe that AI can snatch humans’ job and cause a lot of unemployment (Huang & Rust, 2018), which was one of the most important reasons why many people believe AI was a threat. Therefore, the research of AI threats is of great importance to understand people’s acceptance and use of AI.

Based on the importance of AI threats, Kieslich et al. (2021) developed the Threats of Artificial Intelligence Scale (TAI) to measure the individuals’ perception of AI threat. The TAI hypothesized that AI threat perception is reflected differently in different AI application fields. That is, threat perception is dependent on the AI scenario that the individual encounters (Kieslich et al. 2021). Although it is impossible to exhaust all scenarios, existing research showed that AI has been widely used in the fields of loan origination, job recruitment, and medical treatment. For example, previous research reported that AI approaches to financial problems are more accurate than traditional statistical methods (Bahrammirzaee, 2010). Cardiovascular medicine uses AI to explore new genotypes and phenotypes in existing diseases, hence improving the quality of patient care and reducing readmission and mortality rates (Krittanawong et al., 2017). Furthermore, using AI systems to select potential job applicants is economical and efficient (Tambe et al., 2019). To achieve specific context application fields, Kieslich et al. (2021) developed formal items with clozes for the specific thematic foci, which can be seen as a toolbox that is customizable for distinct areas of application. The final version of the TAI includes three subversions: loan origination, job recruitment, and medical treatment. Each subversion has 12 items, which were divided into four dimensions (recognition, recommendation, decision-making, and prediction). To the best of our knowledge, the TAI is the first available tool in measuring individuals’ threat perception of AI and has demonstrated good validity and reliability through CFA, providing a measurement for predicting threat to AI in different scenes.

Unfortunately, the above-reviewed measurements have been limited to the western context and there currently lacks AI threat perception measures in China. This is concerning especially given the rapidly developing utilization of AI in China. Therefore, the development of a Chinese version of the AI threat scale is vital for Chinese human–computer interaction researchers. The current study aims to adapt and validate the Chinese version of TAI to provide a reliable and valid measurement of the threats of AI for Chinese people. The values for adapting TAI into Chinese are twofold. First, The TAI was developed based on three commonly used application scenes of AI, namely loan origination, job recruitment, and medical treatment, and hence provides a wide application range and all areas highly relevant in China. Second, some researchers found that attitudes toward robots and autonomous systems may vary among different cultures (Gnambs & Appel, 2019). As the only measurement tool for AI threats, adapting and validating a Chinese version of the TAI can not only provide Chinese researchers with a valuable research and practical tool, but also would make cross-cultural comparison of AI threats possible.

To verify the validity of the threat, this study also used other threat-related variables as criteria. For instance, previous studies have reported the relationship between threat and emotions, including positive and negative emotions. Specifically, when the individual feels threatened, the individual’s positive emotions decrease and the negative emotions increase (Bedyńska and Żołnierczyk-Zreda, 2015; Bilandzic et al., 2017; Chen et al., 2020; Constantin & Cuadrado, 2021). Therefore, positive emotions and negative emotions were used as the criteria of threat in this study. In addition, previous research has fully demonstrated that there is a positive relationship between threat and anxiety (Albuquerque et al., 2017; Bar-Haim et al., 2010; Osborne, 2007; Tempel & Neumann, 2014), and individuals with different anxiety levels have different threat biases, that is, threat biases may only be present for high anxious individuals (Berggren & Eimer, 2021). Therefore, this study used anxiety as the criterion variable to further evaluate the criterion validity of the Chinese version of the TAI.

In summary, the aim of the present study was to adapt the TAI into Chinese, and to establish the psychometric properties of the Chinese TAI.

## Method

### Participants

#### Sample 1

We randomly recruited 660 residents in China. Six outliers (operationalized as scores beyond three standard deviations from the mean) were identified, resulting in a final sample of 654 participants. Item analysis and EFA were performed with sample 1. Table 1 presented the demographic information.

### Sample 2

We randomly recruited another 1500 residents in China. Similarly, 17 outliers were evidence, and 1483 participants were remained. Sample 2 was used for CFA and criterion-related validity analysis. Table 1 shows the demographic characteristics.

**Table 1:** Demographic characteristic of samples 1 and 2

	Sample 1		Sample 2	
Variables	*N*	%	*N*	%
Gender				
Male	376	57.49	590	39.78
Female	273	41.74	661	44.57
Others	5	0.76	232	15.64
Age				
< 18	42	6.42	54	3.64
18–25	258	39.45	305	20.57
26–34	253	38.69	549	37.02
35–54	85	13	283	19.08
55–64	10	1.53	111	7.49
≥ 65	6	0.92	181	12.22
Occupational information				
Student	143	21.87	146	9.85
Salesman	158	24.16	349	23.53
Professional (e.g., accountants, lawyers, medical staff)	214	32.72	469	31.63
Manager	97	14.83	213	14.36
Housekeeper	27	4.13	93	6.27
Others	15	2.29	213	14.36
**Highest academic credentials**				
High school or below	93	14.22	148	9.98
College	231	35.33	502	33.85
Bachelor degree	263	40.21	553	37.29
Graduate degree or above	67	10.25	280	18.88

### Procedure

The current study was approved by the Institutional Review Board of the Institute of the Psychology, Zhejiang Sci-Tech University. Prior to data collection, we have obtained permission to use and adapt the TAI from the original authors. According to the power analysis for the two-tailed correlative relationship, type I error rate at 1%, type II error rate at 20%, expected correlative effect size at 0.10, and at least 1160 participants should be included. Additionally, the recommendation of sample to the variable ratio of 10:1 (Nunnally & Bernstein, 1994), the sample size of 760 participants was sufficient to ensure the stability of the results. In conclusion, the sample size in this research had sufficient statistical power.

Participants were recruited through WJX (www.wjx.cn), a Chinese online platform for questionnaire data collection. Data collection was ongoing from November to December 2021. All participants started the survey after reviewing and agreeing to the informed consent. After completing the questionnaire, participants were rewarded with 1.7 Chinese Yuan (CNY). All participants were aware of their right to withdraw from the survey at any time. All collected responses were analyzed in a de-identified manner.

### Measurements

#### The Threats of Artificial Intelligence Scale (TAI)

The threats of AI was measured by the TAI (36 items), including three subversions (loan origination, job recruitment, medical treatment), and each subversions contained 12 items with four dimensions (recognition, recommendation, decision-making, and prediction) (Kieslich et al., 2021). Sample items include “If you now think of the use of AI in recognition, how threatening do you think computer applications of AI are that detect probability of default of credits/creditworthiness?”, “If you now think of the use of AI in recognition, how threatening do you think computer applications of AI are that record probability of default of credits/creditworthiness?” Participants answered on a five-point Likert scale from 1 = “not at all” to 5 = “very strong,” with higher scores indicating higher perceived threat levels.

We took the following steps to translate the TAI into Chinese (Regmi et al., 2010). First, the TAI was translated into Chinese by two psychology postgraduates independently. We compared the two Chinese versions and formed an initial version. Then, the Chinese version of the TAI was translated back into English by two psychologists with professional translation experiences. We compared the differences between the two English versions and the original version to check the accuracy, fluency and adaptation. After that we made necessary modifications to particular words and phraseologies of the Chinese version. Subsequently, the Chinese versions of the TAI was evaluated by a focus group of eight individuals who are randomly selected. These individuals were asked to rate the understandability of each item and offered further suggestions to improve the face validity of the scale. Based on this process, the Chinese version of the TAI was formed.

### Positive and Negative Affect Scale (PANAS)

The PANAS was developed by Gamst et al. (1988), which was used to evaluate the positive and negative emotion of individuals. The Chinese version of PANAS was revised by Huang et al. (2003), including 20 items (e.g., “interested” “upset”) that form two subscales (positive and negative emotion). Participants rated each item on a five-point Likert scale (1 = “not at all” to 5 = “very strong”). The higher the mean score in the positive subscales, the higher levels of positive emotion. The higher the mean score in the negative subscales, the higher levels of negative emotion. In this survey, the Cronbach’s α of the two subscales were 0.85 and 0.83.

### Self-Rating Anxiety Scale (SAS)

The anxiety was measured by the 20-item SAS (Zung & William, 1971). Participants answered on a 5-point Likert scale, from 1 = “not at all” to 5 = “very strong”. Higher scores mean a higher level of anxiety. In this survey, the Cronbach’s α was 0.96.

### Data analysis

**Fig. 1:**
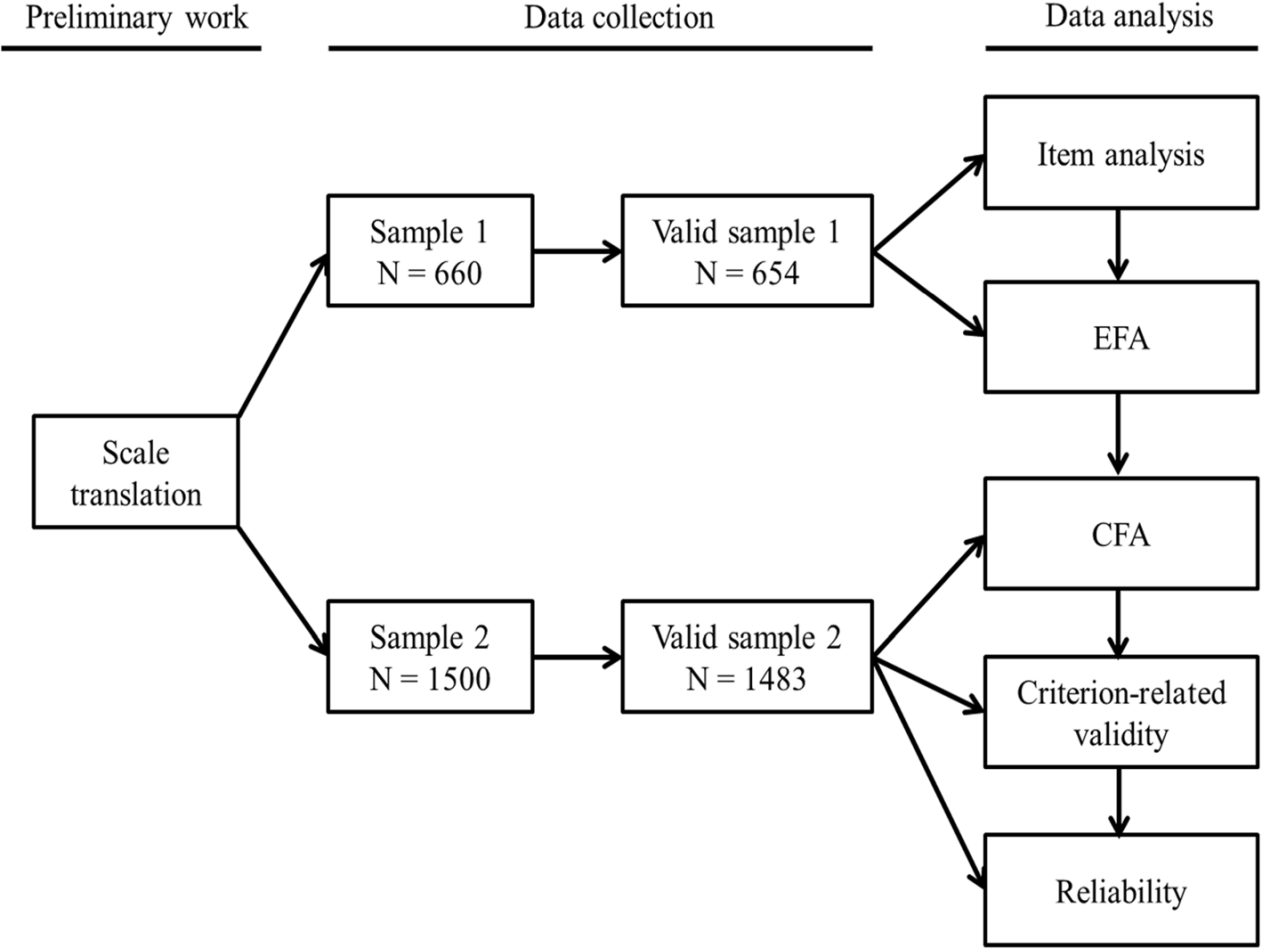
Data analysis framework

Data analysis was conducted in two phases. In the first phase, first, we conducted item analysis with sample 1 to assess the quality of each item. Then, exploratory factor analysis (EFA) was used to explore the structure of the factors and examine the factor loadings of each item to determine whether it needs to be deleted. Finally, reliability analysis was performed in terms of Cronbach’s alpha and Guttman split-half reliability. In the second phase, first, we performed confirmatory factor analysis (CFA) with sample 2 to verify the structure of the factors, then Pearson correlation to analyze the correlation between the criterion variables and AI threats to determine the criterion-related validity. Finally, reliability of the TAI was evaluated in terms of Cronbach’s alpha and Guttman split-half reliability. Moreover, as the TAI scale involves the application of AI in three fields, namely, loan origination, job recruitment and medical treatment. It measures the level of AI threat in these three different fields independently. Each field is relatively independent and measured by different items. As thus, we evaluated the three fields with different model. All the statistical analyses were conducted using via SPSS 18 and Amos 21. The framework of the analysis was shown in the Fig. 1 below.

## Results

### Item analysis

Item analysis was conducted to evaluate the quality of each item. First, the item-total correlation of each item was calculated, demonstrating that all items met the criterion of 0.30 (Nunnally & Bernstein, 1994). Additionally, we selected the highest 27% as the high-score group and the lowest 27% as the low-score group to conduct a *t* test to investigate the discrimination index representing the difference between the high-score group and the low-score group for each item. The results showed that the difference between the two groups for each item was significant (*p*s < 0.01). Overall, the results of item analysis indicated acceptable quality of all items. The results were shown in Table 2.

**Table 2:** Item analysis table

	Loan origination		Job recruitment		Medical treatment	
	*t*	*r*	*t*	*r*	*t*	*r*
Q1	24.49^**^	.78^**^	27.94^**^	.82^**^	29.24^**^	.82^**^
Q2	26.52^**^	.83^**^	24.38^**^	.81^**^	24.68^**^	.80^**^
Q3	24.71^**^	.83^**^	25.91^**^	.82^**^	27.12^**^	.84^**^
Q4	26.96^**^	.84^**^	26.32^**^	.82^**^	24.74^**^	.80^**^
Q5	26.03^**^	.84^**^	24.05^**^	.79^**^	28.65^**^	.82^**^
Q6	26.86^**^	.84^**^	29.02^**^	.84^**^	26.56^**^	.82^**^
Q7	24.92^**^	.83^**^	25.93^**^	.82^**^	28.71^**^	.83^**^
Q8	27.18^**^	.84^**^	25.52^**^	.82^**^	25.83^**^	.83^**^
Q9	26.28^**^	.84^**^	27.01^**^	.81^**^	26.58^**^	.83^**^
Q10	25.89^**^	.83^**^	26.83^**^	.81^**^	27.62^**^	.81^**^
Q11	26.66^**^	.83^**^	26.39^**^	.82^**^	27.31^**^	.83^**^
Q12	24.55^**^	.82^**^	25.83^**^	.82^**^	25.67^**^	.81^**^

*Note*: **p < *.* 01. *r* denotes item-total correlation. *t* denotes the discrimination index

### Exploratory factor analysis

First, the Kaiser–Meyer–Olkin (KMO) value was between 0.96 and 0.97, and results of Bartlett’s sphericity tests all reached statistical significance (*p*s < 0.001), indicating that the sample was suitable for EFA. Second, we adopted a parallel analysis to determine the number of factors (Hayton et al., 2004) and found that a single factor model was supported for each subversion. Subsequent principal component method was used to extract a single factor for each subversion, providing eigenvalues ranging from 8.55 to 8.82, total interpretation rate of variance ranging from 71.26 to 73.50%, and all loadings of the items being greater than 0.65. The loadings for each item were shown in Table 3.

**Table 3:** Results of exploration factor analysis (*n* = 654)

	Loan origination	Job recruitment	Medical treatment
Q1	.81	.83	.86
Q2	.82	.85	.86
Q3	.84	.85	.85
Q4	.85	.85	.86
Q5	.87	.85	.85
Q6	.85	.87	.86
Q7	.85	.86	.86
Q8	.86	.85	.87
Q9	.84	.85	.86
Q10	.86	.86	.85
Q11	.85	.84	.85
Q12	.85	.85	.85
total	8.55	8.69	8.82
% of variance	71.26	72.44	73.50
KMO	.96	.97	.97

### Confirmatory factor analysis

The model fit indices for CFA were evaluated against the following criteria: comparative fit index (CFI) value, relative fit index (RFI) value, incremental fit index (IFI) value and Tucker-Lewis index (TLI) value are equal or greater than 0.90, root mean square error of approximation (RMSEA) values and standardized root mean squared residual (SRMR) values equal or less than 0.08, and the *χ*^2^/*df* values of up to 3 (Hu & Bentler, 1999).

### Loan origination

The result of CFA showed that *χ*^2^ = 1200.29, df = 54, *p* = 0.00, *χ*^2^/df = 22.23, CFI = 0.93, TLI = 0.92, RFI = 0.91, IFI = 0.93, RMSEA = 0.120, SRMR = 0.03, indicating that the one-factor structure of the Chinese version of the TAI was fitting acceptable in the loan origination subversion. The items loadings were between 0.78 and 0.85.

### Job recruitment

The result of CFA showed that *χ*^2^ = 1167.39, df = 54, *p* = 0.00, *χ*^2^/df = 21.62, CFI = 0.93, TLI = 0.92, RFI = 0.92, IFI = 0.93, RMSEA = 0.121, SRMR = 0.04, indicating that the one-factor structure of the Chinese version of the TAI was fitting acceptable in the job recruitment subversion. The item loadings were between 0.81 and 0.85.

### Medical treatment

The result of CFA showed that *χ*^2^ = 1088.91, df = 54, *p* = 0.00, *χ*^2^/df = 20.17, CFI = 0.94, TLI = 0.93, RFI = 0.92, IFI = 0.94, RMSEA = 0.116, SRMR = 0.03, indicating that the one-factor structure of the Chinese version of the TAI was fitting acceptable in the medical treatment subversion. The items loadings were between 0.83 and 0.86.

### Criterion-Related Validity

Pearson correlation analysis was used to analyze the criterion-related validity. It can be found that, in the field of loan origination, TAI has a high positive relationship with negative affect (*r* = 0.28^**^, *p* < 0.01), anxiety (*r* = 0.19^**^, *p* < 0.01), and a negative relationship with positive affect (*r* =  − 0.32^**^, *p* < 0.01). In the field of job recruitment, TAI has a high positive relationship with negative affect (*r* = 0.25^**^, *p* < 0.01), anxiety (*r* = 0.17^**^, *p* < 0.01), and a negative relationship with positive affect (*r* =  − 0.25^**^, *p* < 0.01). Moreover, in the field of medical treatment, TAI has a high positive relationship with negative affect (*r* = 0.21^**^, *p* < 0.01), anxiety (*r* = 0.14^**^, *p* < 0.01), and a negative relationship with positive affect (*r* =  − 0.23^**^, *p* < 0.01). These results indicate that the Chinese version of TAI demonstrated good criterion-related validity.

### Reliability analysis

The results showed that these coefficients are all high, which indicates that the scale has high reliability in each field (Table 4).

**Table 4:** Reliability coefficient

Field	Sample 1			Sample 2	
	Cronbach’s α	Guttman split-half reliability		Cronbach’s α	Guttman split-half reliability
Loan origination	.96		.94	.96	.95
Job recruitment	.95		.94	.97	.96
Medical treatment	.96		.95	.97	.97

## Discussion

The purpose of this study was to adapt the TAI into Chinese and establish its reliability and validity in China. As an effective tool for evaluating the threat of AI, the Chinese version of the TAI would be useful in exploring the characteristics of public concerns about the use of AI systems in China and the related influencing factors. Our results indicated that the Chinese version of the TAI is a valid tool to measure threat of AI in China.

Staying consistent with the test adaption procedure as utilized in previous studies (Cai et al., 2022; Li et al., 2021), we first used EFA to examine the structure of the scale. The results revealed that only one dimension was extracted in this study. To further verify the factor structure, we also performed a series of CFA analyses. It was found that the model fitting of the single factor was high, which further verified the validity of the factor structure. Of note, the one-factor structure within each dimension differs from the English version of original scale that included four dimensions. This may be related to different cultural perspectives. Individuals from a western culture likely think analytically and tend to analyze things step-by-step, whereas those from eastern cultures are more likely to think holistically (Morris & Peng, 1994; Peng & Nisbett, 1999). Therefore, as a result, individuals likely view all items as the same dimension in China, while the westerners view them as different dimensions. In addition, it is also possible that AI is a relatively new technology in China, making individuals not explicitly distinguishing AI functions just yet.

To further provide evidence of criterion-related validity of the TAI, the current study examined the relationship between AI threat and emotion, anxiety. Results revealed a significant negative correlation between positive emotion and threat, and a significant positive correlation between negative emotion, anxiety, and threat. Previous research showed that threat is a good predictor of negative emotions (Chen et al., 2020). That is, when an individual encounters something that threatens him/her, the individual is prone to negative emotions. Since positive emotions and negative emotions are opposite emotions, this study also found a significant negative correlation between positive emotions and threats. Additionally, previous research showed that threat can affect individual’s anxiety (Albuquerque et al., 2017), and anxious individuals have a higher attentional bias for threats (Berggren & Eimer, 2021; Zhao et al., 2014). The results of this study are consistent with those of previous studies, that is, the individual feels threatened when interacting with the AI, the individual will be more prone to anxiety. In conclusion, this study revealed the effectiveness of emotion and anxiety as criterions of AI threat in the Chinese context.

This study has three contributions. First, the introduction of the Chinese version of the TAI provides a tool for examining the threat of AI in Chinese culture, which can promote cross-cultural understanding and help clarify the characteristics of threat perception in non-English cultures. Moreover, the previous research mainly focused on the cognitive assessment of AI interaction, such as AI trust (Chi et al., 2021), attitude (Schepman et al., 2022). The TAI scale focuses on the emotional assessment of AI interaction, and the Chinese TAI provides a research basis for the emotion assessment of AI interaction in the future. Finally, the Chinese version of the TAI verified the relationship between positive and negative emotions, anxiety and threat perception of AI in the Chinese context.

Some limitations should be acknowledged. First, we recruited a large sample from eastern and southern China, and the generality of the results was limited. The validity and reliability of the Chinese version of the TAI in northern and western China need further study. Second, we choose two tools to test the validity of the Chinese TAI. More research should use other tools to better understand the characteristics and strengths, as well as weaknesses of the Chinese TAI. Thirdly, the Cronbach’s α and the split-half reliability coefficient were used in this study. In future studies, test–retest reliability should be considered to test the reliability of Chinese version of the TAI. Finally, we investigated different demographic information in this study, such as occupational information. More research needs to examine the applicability of the Chinese TAI in different groups.

## Conclusion

This study examined the factor structure, reliability and criterion-related validity of the Chinese TAI. We confirmed that the Chinese TAI had good psychometric properties. Specifically, first, the Chinese TAI had a clear factor structure, and the fitting index were acceptable. Second, the Chinese TAI had a high Cronbach’s α coefficient and Guttman split-half reliability. Finally, the Chinese TAI had a high positive relationship with negative affect, anxiety, and a negative relationship with positive affect, indicating that the Chinese TAI had a high criterion-related validity. In sum, the Chinese TAI is a valid and reliable measurement to evaluate threat perception towards AI.

## Data Availability

The data that support the findings of this study are available on request from the corresponding author. The data are not publicly available due to privacy or ethical restrictions.

## Abbreviations

TAI: Threats of artificial intelligence scale
AI: Artificial intelligence
EFA: Exploratory factor analysis
CFA: Confirmatory factor analysis
PANAS: Positive and Negative Affect Scale
SAS: Self-Rating Anxiety Scale
KMO: Kaiser-Meyer-Olkin
CFI: Comparative fit index
RFI: Relative fit index
IFI: Incremental fit index
TLI: Tucker-Lewis index
RMSEA: Root mean square error of approximation
SRMR: Standardized root mean squared residual
